# Current practice of antibiotic prophylaxis for surgical fixation of closed long bone fractures: a survey of 297 members of the Orthopaedic Trauma Association

**DOI:** 10.1186/s13037-016-0118-5

**Published:** 2017-01-16

**Authors:** Itai Gans, Amit Jain, Norachart Sirisreetreerux, Elliott R. Haut, Erik A. Hasenboehler

**Affiliations:** 1 Department of Orthopaedic Surgery, The Johns Hopkins University, 601 N Caroline Street, Baltimore, MD 21287 USA; 2 Department of Surgery, Division of Acute Care Surgery, The Johns Hopkins Hospital, 600 N Wolfe Street, Baltimore, MD 21287 USA

**Keywords:** Antibiotic prophylaxis, Long bone fractures, Orthopaedic trauma, Perioperative antibiotics, Surgical site infection

## Abstract

**Background:**

The risk of postoperative surgical site infection after long bone fracture fixation can be decreased with appropriate antibiotic use. However, there is no agreement on the superiority of a single- or multiple-dose perioperative regimen of antibiotic prophylaxis. The purpose of this study is to determine the following: 1) What are the current practice patterns of orthopaedic trauma surgeons in using perioperative antibiotics for closed long bone fractures? 2) What is the current knowledge of published antibiotic prophylaxis guidelines among orthopaedic trauma surgeons? 3) Are orthopaedic surgeons willing to change their current practices?

**Methods:**

A questionnaire was distributed via email between September and December 2015 to 955 Orthopaedic Trauma Association members, of whom 297 (31%) responded.

**Results:**

Most surgeons (96%) use cefazolin as first-line infection prophylaxis. Fifty-nine percent used a multiple-dose antibiotic regimen, 39% used a single-dose regimen, and 2% varied this decision according to patient factors. Thirty-six percent said they were unfamiliar with Centers for Disease Control and Prevention (CDC) antibiotic prophylaxis guidelines; only 30% were able to select the correct CDC recommendation from a multiple-choice list. However, 44% of surgeons said they followed CDC recommendations. Fifty-six percent answered that a single-dose antibiotic prophylaxis regimen was not inferior to a multiple-dose regimen. If a level-I study comparing a single preoperative dose versus multiple perioperative antibiotic dosing regimen for treatment of closed long bone fractures were published, most respondents (64%) said they would fully follow these guidelines, and 22% said they would partially change their practice to follow these guidelines.

**Conclusion:**

There is heterogeneity in the use of single- versus multiple-dose antibiotic prophylaxis for surgical repair of closed long bone fractures. Many surgeons were unsure of current evidence-based recommendations regarding perioperative antibiotic use. Most respondents indicated they would be receptive to high-level evidence regarding the single- versus multiple-dose perioperative prophylactic antibiotics for the treatment of closed long bone fractures.

**Electronic supplementary material:**

The online version of this article (doi:10.1186/s13037-016-0118-5) contains supplementary material, which is available to authorized users.

## Background

Surgical site infections (SSIs) account for approximately 38% of all postoperative infections and can be devastating after treatment of long bone fractures [1]. Rates of SSI after surgery for closed fractures range from 1 to 4% [2, 3]. Perioperative antibiotic prophylaxis can help prevent SSI, and studies have shown the benefit of administering antibiotics immediately before skin incision in closed fracture surgery [4–11].

Antibiotic administration before surgical incision has markedly improved the safety of modern surgery, especially in cases with high risk of infection, including the use of retained prosthetic instrumentation as is used in closed long bone fracture repair [12]. Although antibiotic administration before incision is the standard of care [9], there is no agreement on the appropriate perioperative duration of antibiotic prophylaxis. The Centers for Disease Control and Prevention (CDC) Infection Control and Hospital Epidemiology guidelines published in 1999 recommend that antibiotics be re-administered if the duration of surgery is expected to exceed the time during which therapeutic levels of the antibiotic can be maintained and, at most, until a few hours after the surgery has ended [1]. These guidelines, however, do not indicate whether postoperative continuation of antibiotic prophylaxis is necessary. Appropriate dosing is necessary to ensure the agent’s concentration exceeds the minimal bactericidal concentration against the target pathogen at the surgical site for the duration of surgery [13]. In fact, the American Academy of Orthopaedic Surgeons Committee on Patient Safety published guidelines in 2009 [14, 15] recommending perioperative antibiotic treatment to include a single preoperative dose and intraoperative re-dosing based on procedure length and blood loss and to discontinue antibiotics within 24 h after wound closure; however, the recommendations do not specify whether a single preoperative antibiotic dose or a 24-h prophylaxis regimen is recommended. The choice of single- versus multiple-dose perioperative antibiotics is influenced by factors such as injury severity, comorbidities, and duration of surgery. Recent reviews have highlighted the controversy that exists in the literature in selection of an antibiotic dosing regimen for prevention of surgical site infection; although there is widespread use, there is no agreed-upon agent or dosing duration that has been found to be statistically superior [13, 16, 17].

We hypothesized that there would be heterogeneity in practice patterns among orthopaedic trauma surgeons in terms of using single- versus multiple-dose antibiotic regimens. We also hypothesized that many orthopaedic trauma surgeons would be unfamiliar with published antibiotic prophylaxis guidelines but would be willing to change their practices to help prevent SSI. This study assessed the following questions: 1) What are the current practice patterns of members of the Orthopaedic Trauma Association (OTA) in using perioperative antibiotics for closed long bone fractures? 2) What is the current knowledge of published antibiotic prophylaxis guidelines among orthopaedic trauma surgeons? 3) Are orthopaedic surgeons willing to change their current antibiotic prophylaxis practices?

## Methods

After approval from our institutional review board and the OTA, an electronic survey (Additional file 1) was conducted using Google Forms software (Google Corporation, Mountain View, CA). The survey questions were written by the senior author, who is a practicing traumatologist, and by the research investigators, who are orthopaedic surgery residents. The questions were refined on the basis of feedback from an internal panel of traumatologists for face validity. Survey questions assessed the perioperative antibiotic practices of surgeons treating closed long bone fractures surgically, as well as the surgeons’ familiarity with recommendations regarding perioperative antibiotic use. The survey was distributed to members of the OTA between September and December 2015 through a posting on the OTA website and through email. Follow-up email reminders were sent 4 and 8 weeks after the initial invitation. The survey remained open for 12 weeks. Participating surgeons were anonymous and participation was voluntary. Respondents were not compensated. Of the 955 OTA members, 297 (31%) responded to the survey at the time of its closure.

### Survey design

The survey consisted of three sections. Section 1 inquired about the number of years the respondent had spent in post-training clinical practice and the number of closed long bone fractures the respondent had treated in the previous year. Section 2 inquired about the surgeon’s current practice of using perioperative antibiotics. Respondents were asked to assume treatment of a patient with a closed long bone fracture requiring surgical fixation with no soft-tissue or other related injury. They were asked to assume a surgical duration of less than 4 h from anesthesia to wound closure in a patient with no known medication allergies, no history of methicillin-resistant *Staphylococcus aureus* or vancomycin-resistant *Enterococcus* infection, and no immunocompromised state. Questions in section 2 were multiple choice with the option of an open-ended response. Section 3 inquired about respondents’ knowledge of current perioperative antibiotic guidelines for orthopaedic closed long bone surgery. Questions were true/false or multiple choice. All questions had an option to answer “unsure.” Lastly, respondents were asked in a multiple-choice format if they would change their current perioperative antibiotic practices if a study providing level-I evidence regarding use of perioperative antibiotics in orthopaedic trauma were published.

### Data analysis

Survey data were analyzed by two members of our study team. Incomplete surveys were excluded. We used Microsoft Excel, version 2016, software (Microsoft, Redmond, WA) to summarize the responses to all survey questions and present descriptive statistics.

## Results

A total of 297/955 (31%) members of the OTA completed the survey. Three incomplete surveys were received and were not included in the results of this study. Of the respondents, 21% had 0 to 5 years of post-training clinical practice, 23% had 6 to 10 years, 13% had 11 to 15 years, 19% had 16 to 20 years, 11% had 21 to 25 years, 9.1% had 26 to 30 years, and 6.1% had ≥31 years. The median number of long bone fractures treated annually per surgeon was 180 (range, 10–1000).

### Current practice patterns

Most respondents (96%, *n* = 284) used cefazolin as their preferred first-line prophylaxis antibiotic for treatment of closed long bone fractures (Table 1). Others used cefuroxime, flucloxacillin and gentamicin together, ceftriaxone, clindamycin, vancomycin, cefazolin and vancomycin together, or did not use antibiotics. Most respondents (58%, *n* = 171) administered one dose of antibiotic within 1 h prior to incision and re-administered for 24 h (Table 2). Some respondents (19%, *n* = 56) administered one dose of antibiotic within 30 min prior to incision and did not re-administer postoperatively. Others (14%, *n* = 42) administered one dose within 1 h prior to incision and did not re-administer postoperatively. Others’ practices varied situationally, with 2% (*n* = 6) of respondents reporting varying their practices according to injury severity, fracture type, and operative intervention.

**Table 1:** Preferred first-line surgical prophylactic antibiotic regimen for treatment of closed long bone fractures determined by a survey of 297 Orthopaedic Trauma Association members, September–December, 2015

Preferred antibiotic	No. (%) of surgeons
Cefazolin	284 (96)
Cefuroxime	5 (1.7)
No antibiotics	2 (0.7)
Flucloxacillin and gentamicin	2 (0.7)
Ceftriaxone	1 (0.3)
Clindamycin	1 (0.3)
Vancomycin	1 (0.3)
Cefazolin and vancomycin	1 (0.3)

**Table 2:** Preferred prophylactic antibiotic re-dosing regimen for treatment of closed long bone fractures determined by a survey of 297 Orthopaedic Trauma Association members, September–December, 2015

Preferred dosing regimen	No. (%) of surgeons
One dose within 15 min prior to incision only	18 (6.1)
One dose within 30 min prior to incision only	56 (19)
One dose within 60 min prior to incision only	42 (14)
One dose within 60 min prior to incision and 24-h re-dosing	171 (58)
One dose within 60 min prior to incision and 48-h re-dosing	2 (0.7)
One dose within 60 min prior to incision and 1 postoperative dose	2 (0.7)
Varied by injury severity, fracture type, and operative intervention	6 (2.0)

When asked how frequently they re-administer antibiotics during a procedure, 63% of respondents (*n* = 188) said they re-administered every 4 h. Others re-administered every 3 h (22%, *n* = 65), 5 h (1.3%, *n* = 4), or 6 h (1%, *n* = 3). Ten percent of respondents (*n* = 30) said they did not re-administer antibiotics during surgery. Others (*n* = 7) said they re-administered antibiotics on the basis of estimated blood loss or antibiotic half-life.

Of the 263 respondents who re-administered antibiotics intraoperatively (with a total of 320 responses because some respondents had multiple reasons), 75% (*n* = 198) re-administered according to the half-life of the antibiotic or duration of surgery, and 20% (*n* = 54) re-administered according to estimated blood loss or intravenous fluid dilution as the case progressed. Others cited literature to support their dosing patterns or re-administered according to hospital guidelines, wound size, procedure type, or complexity of the case. Only three respondents (1.1%) re-administered before making a second incision in more complex surgical cases (Table 3).

**Table 3:** Reasons given by 263 surgeons who re-administered antibiotics intraoperatively, according to a survey of Orthopaedic Trauma Association members, September–December, 2015

Reason	No. (%) of surgeons
Half life/duration of surgery	198 (75)
Estimated blood loss/IV fluid dilution	54 (21)
Hospital guidelines	24 (9.1)
Referred to literature	19 (7.2)
Wound size, procedure type, case complexity	15 (5.7)
Ambient instrument contamination because of case length	7 (2.7)
Making a second incision	3 (1.1)

### Knowledge of evidence

When asked if a single-dose of long-acting intravenous antibiotic was inferior to any multiple-dose antibiotic regimen to prevent orthopaedic SSIs, 56% (*n* = 165) of respondents answered false, 5.4% (*n* = 16) answered true, and 39% (*n* = 116) were unsure. When asked if a multiple-dose antibiotic regimen (i.e., coverage for 24 h) significantly reduced the risk of SSIs compared with a single dose of preoperative antibiotics, 62% (*n* = 185) of respondents answered false, 8.8% (*n* = 26) answered true, and 29% (*n* = 86) were unsure.

When asked how frequently prophylactic antibiotics should be administered intraoperatively to maintain minimum inhibitory concentration, 5.1% (*n* = 15) of respondents answered every 2 to 3 h, 36% (*n* = 106) answered every 4 to 6 h, 1.7% (*n* = 5) answered every 6 to 12 h, 26% (*n* = 77) answered when the duration of the procedure exceeds 1 to 2 times the half-life of the antibiotic, 2.7% (*n* = 8) answered when the duration of the procedure exceeds 3 to 4 times the half-life of the antibiotic, and 29% (*n* = 85) were unsure.

When surgeons were asked which answer choice had category IA evidence to support its recommendation regarding perioperative antibiotic usage and dosing based on the *Guideline for Prevention of Surgical Site Infection* published by the CDC in 1999 [18], 30% (*n* = 90) answered correctly: “maintain therapeutic levels of the antibiotic agent in serum and soft tissue throughout the operation and until, at most, a few hours after the incision was closed in the operating room.” Another 2.7% (*n* = 8) answered “maintain therapeutic levels of the antibiotic agent in serum and soft tissues throughout the operation and until 12 h after the incision is closed in the operating room”; 31% (*n* = 92) answered “maintain therapeutic levels of the antibiotic agent in serum and soft tissues throughout the operation and until 24 h after the incision is closed in the operating room”; 0% (*n* = 0) answered “maintain therapeutic levels of the antibiotic agent in serum and soft tissues throughout the operation and until 48 h after the incision is closed in the operating room”; and 36% (*n* = 107) were unsure of the published guideline. When asked if they follow this CDC guideline to prevent SSI, 44% (*n* = 132) said yes, 8.8% (*n* = 26) said no, 41% (*n* = 123) answered that they did not know the CDC-recommended dosing, and 5.4% (*n* = 16) preferred not to answer.

### Willingness to change practice patterns

If a level-I study comparing a single preoperative dose versus multiple perioperative antibiotic dosing regimen for treatment of closed long bone fracture were published, 191 respondents (64%) said they would fully follow these guidelines. Sixty-five respondents (22%) said they would somewhat follow the evidence but adjust dosing on a case-by-case basis; 7 respondents (2.4%) said they would continue their current practices, 19 respondents (6.4%) said they would follow their hospital’s guidelines rather than published guidelines, and 15 respondents (5.1%) said they were unsure if they would make a change to their current practices.

## Discussion

We found heterogeneity in the use of single- versus multiple-dose antibiotics by orthopaedic trauma surgeons treating closed long bone fractures, with 39% administering only 1 preoperative dose and 59% using at least 1 additional postoperative dose. We also found that many respondents were unfamiliar with published antibiotic prophylaxis guidelines. Despite familiarity with recommendations that show no benefit of postoperative antibiotic prophylaxis, many surgeons continue to administer antibiotics postoperatively. Respondents said they would be willing to change their practices if a well-performed study were published regarding the use of perioperative antibiotics in closed orthopaedic trauma cases.

Clinical practice guidelines for perioperative antibiotic prophylaxis developed jointly by the American Society of Health-System Pharmacists, the Infectious Diseases Society of America, the Surgical Infection Society, and the Society for Healthcare Epidemiology of America recommend routine use of cefazolin unless contraindicated for “clean” orthopaedic procedures involving internal fixation such as the treatment of closed long bone fractures [19]. In the current study, 96% of surgeons used cefazolin as their preferred preoperative antibiotic prophylaxis. This shows a high rate of compliance with recommendations. In the current study, 85% of respondents reported they re-administer antibiotics every 3 to 4 h, and 96% of respondents reported using cefazolin, indicating high compliance with the CDC recommendation to maintain therapeutic levels of antibiotics throughout the procedure. Recent meta-analyses [13, 20, 21] have failed to show the superiority of multiple-dosing antibiotic regimens versus a single preoperative dose in terms of preventing both superficial and deep SSI, and to our knowledge, there are no established clinical practice guidelines on this topic. Despite this, our study shows that many orthopaedic surgeons continue to use multiple-dose antibiotic prophylaxis when treating closed fractures. In 2009, the American Academy of Orthopaedic Surgeons Committee on Patient Safety released perioperative antibiotic prophylaxis guidelines that include using a single preoperative antibiotic dose, re-dosing antimicrobials intraoperatively for prolonged procedures or in patients with significant blood loss, and discontinuing antibiotics within 24 h after wound closure when using postoperative doses [14, 15]. However, there are no recommendations to help orthopaedic surgeons decide whether to continue antibiotics postoperatively. In 2008 [14], a literature review found that antimicrobial prophylaxis after wound closure did not provide additional protection against SSI [22]. Similarly, clinical practice guidelines developed by the American Society of Health-System Pharmacists, the Infectious Diseases Society of America, the Surgical Infection Society, and the Society for Healthcare Epidemiology of America do not definitively recommend a single- or multiple-dose antibiotic prophylaxis regimen. Given this ambiguity, it would be unsurprising for orthopaedic surgeons to have heterogeneous practice patterns.

The CDC’s Infection Control and Hospital Epidemiology guidelines published in 1999 recommend that antibiotics be re-administered if the duration of surgery is expected to exceed the time during which therapeutic levels of the antibiotic can be maintained and, at most, until a few hours after the surgery has ended [1]. These guidelines do not make a recommendation regarding overall duration of antibiotic administration, nor do they indicate whether postoperative administration is necessary. In our survey, approximately one-third of respondents were unfamiliar with the CDC guidelines and only approximately one-third were able to select the correct CDC recommendation from the list of multiple-choice answers provided, indicating a lack of knowledge of current antibiotic prophylaxis guidelines in the orthopaedic trauma community. Nevertheless, when asked if they follow the CDC recommendation, 44% of respondents said they comply, which was greater than the percentage of respondents who could correctly identify the current guideline. Additionally, a Cochrane review [13] and meta-analysis [21] favor a single-dose regimen. In our study, 39% of respondents were unsure whether a multiple-dose regimen was superior to a single-dose regimen. Fifty-six percent of respondents answered that single-dose antibiotic prophylaxis was not inferior to a multiple-dose regimen, but only 39% of respondents used a single-dose regimen. Clinically, the implications of administering a multiple-dose antibiotic regimen when a single-dose regimen would suffice include use of unnecessary antibiotics, which could cultivate antibiotic resistance in certain bacterial strains that are more difficult to treat.

To our knowledge no high-level study has defined guidelines specific to orthopaedic closed long bone fractures. Accordingly, 64% of respondents were in favor of following recommendations that would be supported by a level-I study comparing a single-dose preoperative antibiotic with a multiple-dose perioperative antibiotic regimen. Twenty-two percent said they would “somewhat” change their practice, and only 2.4% said they would not change their practice if such guidelines became available.

This study is limited by factors inherent in the survey methodology, including the risk of nonresponder bias. Certain questions designed to assess respondents’ knowledge of the orthopaedic literature in a true-or-false format may not capture respondents’ actual knowledge of the current orthopaedic literature regarding these topics. There may be self-reporting bias, with respondents possibly answering in a way that is a positive reflection of their practice, and it is difficult to control for respondents looking up answers and references before responding to questions in an online survey. However, respondents were asked to answer survey questions as objectively as possible, and the answer choice “unsure” was available and selected frequently, indicating that many respondents answered according to their current knowledge of the literature. It is difficult to determine whether the demographic characteristics and practice patterns of the responders to this survey reflect those of the orthopaedic community at large. Specifically, practice patterns of members of the OTA may not reflect practice variation within the orthopaedic community. This study has several strengths. First, only members of the OTA who treat primarily fractures were surveyed. Second, the response rate of 31% was high for a survey of the OTA, in which response rates are typically 10 to 15% [23–25]. Third, surgeons were able to enter open-ended responses to some questions, enabling them to indicate that their practice patterns did not fit into the multiple-choice options. Fourth, the electronic format of the survey and brief, concise, questions and survey allowed us to minimize the number of incomplete surveys.

## Conclusions

Our study shows that there is heterogeneity in the use of antibiotic prophylaxis for surgical treatment of closed long bone fractures in terms of single- versus multiple-dose regimens. These variations likely arise from an experience-based approach, given that many survey respondents were unsure of current evidence-based recommendations regarding perioperative antibiotic use. The results indicate that the orthopaedic community would be receptive to high-level evidence regarding the efficacy of a single-dose versus multiple-dose perioperative antibiotic prophylaxis regimen for the treatment of closed long bone fractures. Studies focusing on this comparison are needed.

## Additional file

Additional file 1: Survey sent to Orthopaedic Trauma Association members to determine perioperative antibiotic use patterns for patients with closed long bone fractures, September–December 2015. (DOCX 15 kb)

## Abbreviations

CDC: Centers for disease control and prevention
OTA: Orthopaedic trauma association
SSI: Surgical site infection
