# Supplementary material for: Current practice of antibiotic prophylaxis for surgical fixation of closed long bone fractures: a survey of 297 members of the Orthopaedic Trauma Association
Source: Patient Saf Surg. 2017 Jan 16;11:2. doi: 10.1186/s13037-016-0118-5 (PMC5240258; doi:10.1186/s13037-016-0118-5)
Supplement: Additional file 1: — Survey sent to Orthopaedic Trauma Association members to determine perioperative antibiotic use patterns for patients with closed long bone fractures, September–December 2015. (DOCX 15 kb) [file 13037_2016_118_MOESM1_ESM.docx]

**Additional file 1**

Survey sent to Orthopaedic Trauma Association members to determine perioperative antibiotic use patterns for patients with closed long bone fractures, September–December 2015.

**Section 1:**

1. Number of years in practice (post-training):
   1. 0 to 5 years
   2. 6 to 10 years
   3. 11 to 15 years
   4. 16 to 20 years
   5. 21 to 25 years
   6. 26 to 30 years
   7. 31 or more years
2. Approximate number of closed long bone fracture surgical procedures (Open Reduction and Internal Fixation, nailing, pinning, etc.) performed in the last 1 year:
   1. Open-ended response

**Section 2:**

For all questions below, please assume the patient had a closed long bone fracture requiring surgical fixation with no soft-tissue or other injuries. Assume the surgery takes <4 hours from anesthesia induction to wound closure. Also assume patient has no allergies to medications and does NOT have a history of methicillin resistant staphylococcus aureus or vancomycin resistant enterococcus, and is NOT immunocompromised.

1. What is your preferred first-line infection prophylaxis antibiotic of choice for prevention of deep wound infections post-surgically:
   1. No antibiotic
   2. Cefazolin (Ancef)
   3. Ceftriaxone (Rocephin)
   4. Cefuroxime (Zinacef)
   5. Cefepime
   6. Clindamycin
   7. Vancomycin
   8. Other: Open-ended response
2. What is your preferred dosing regimen for perioperative antibiotic prophylaxis?
   1. No antibiotic
   2. 1 dose <15 minutes prior to incision
   3. 1 dose within 30 minutes prior to incision
   4. 1 dose within 1 hour prior to incision
   5. 1 dose within 1 hour prior to incision + up to 24 hour re-dosing
   6. 1 dose within 1 hour prior to incision + up to 48 hour re-dosing
   7. It depends on patient injury, type of fracture, and operative intervention
   8. Other: Open-ended response
3. How frequently do you re-dose your antibiotic during the procedure?
   1. I do not re-dose antibiotics during the operative procedure
   2. Every 1 to 2 hours during the procedure
   3. Every 3 hours during the procedure
   4. Every 4 hours during the procedure
   5. Every 5 hours during the procedure
   6. Other: Open-ended response
4. Why do you re-dose antibiotics intraoperatively? Please explain briefly (i.e., type of procedure, type of fracture, duration of surgery, type of antibiotic used, knowledge of published recommendations [if so, please name/refer to the article to the best of your ability without searching for it]).
   1. Open-ended response

**Section 3:**

Please answer the following questions based on your current knowledge of the orthopaedic trauma literature. Please answer these questions to the best of your ability without performing a literature search while taking this survey.

1. True or False: A single dose of long-acting IV antibiotic is inferior to any multiple-dose antibiotic regimen in prevention of orthopaedic surgical site deep wound infections?
   1. True
   2. False
   3. Unsure (If you are unfamiliar with this literature, please select this response)
2. True or False: A multiple-dose antibiotic regiment (i.e., coverage for 24 hours) compared with a preoperative single-dose antibiotic prophylaxis is associated with significant reduction in rate of deep wound infections on multivariate analysis?
   1. True
   2. False
   3. Unsure (If you are unfamiliar with this literature, please select this response)
3. How frequently should prophylactic antibiotics be dosed intraoperatively to maintain an appropriate minimum inhibitor concentration?
   1. Every 2 to 3 hours during the procedure
   2. Every 4 to 6 hours during the procedure
   3. Every 6 to 12 hours during the procedure
   4. When the duration of the procedure exceeds 1 to 2 times the half-life of the antibiotic
   5. When the duration of the procedure exceeds 3 to 4 times the half-life of the antibiotic
   6. Unsure (If you are unfamiliar with this literature, please select this response)
4. Based on the “Guideline for Prevention of Surgical Site Infection,” published by the Center for Disease Control and Prevention in 1999, there is category IA evidence to support which of the following for effective prevention of deep wound infections post-surgically in orthopaedic surgery?
   1. Maintain therapeutic levels of the antibiotic agent in serum and soft tissues throughout the operation and until, at most, a few hours after the incision is closed in the operating room
   2. Maintain therapeutic levels of the antibiotic agent in serum and soft tissues throughout the operation and until 12 hours after the incision is closed in the operating room
   3. Maintain therapeutic levels of the antibiotic agent in serum and soft tissues throughout the operation and until 24 hours after the incision is closed in the operating room
   4. Maintain therapeutic levels of the antibiotic agent in serum and soft tissues throughout the operation and until 48 hours after the incision is closed in the operating room
   5. Unsure (If you are unfamiliar with this literature, please select this response)
5. Do you follow this 1999 guideline for prevention of surgical site infection published by the Center for Disease Control and Prevention?
   1. Yes
   2. No
   3. I do not know the recommended dosing proposed in the Center for Disease Control and Prevention guideline
   4. I prefer not to answer this question
6. If a Level-I study comparing single preoperative dose vs. multiple perioperative antibiotic regimen in treatment of closed long bone fractures were to be published with significant findings, how likely would you be to change your future practice?
   1. I would follow the Level-I evidence
   2. I would somewhat follow the evidence, but adjust the antibiotic dosing duration on a case by case basis
   3. I would continue my current perioperative antibiotic dosing practice
   4. I would follow my hospital’s guidelines
   5. Unsure
